# Automatic Detection of Diseased Tomato Plants Using Thermal and Stereo Visible Light Images

**DOI:** 10.1371/journal.pone.0123262

**Published:** 2015-04-10

**Authors:** Shan-e-Ahmed Raza, Gillian Prince, John P. Clarkson, Nasir M. Rajpoot

**Affiliations:** 1 Department of Computer Science, University of Warwick, Coventry, UK; 2 School of Life Sciences, University of Warwick, Coventry, UK; 3 Department of Computer Science and Engineering, Qatar University, Qatar; Julius Kuehn-Institute (JKI), GERMANY

## Abstract

Accurate and timely detection of plant diseases can help mitigate the worldwide losses experienced by the horticulture and agriculture industries each year. Thermal imaging provides a fast and non-destructive way of scanning plants for diseased regions and has been used by various researchers to study the effect of disease on the thermal profile of a plant. However, thermal image of a plant affected by disease has been known to be affected by environmental conditions which include leaf angles and depth of the canopy areas accessible to the thermal imaging camera. In this paper, we combine thermal and visible light image data with depth information and develop a machine learning system to remotely detect plants infected with the tomato powdery mildew fungus *Oidium neolycopersici*. We extract a novel feature set from the image data using local and global statistics and show that by combining these with the depth information, we can considerably improve the accuracy of detection of the diseased plants. In addition, we show that our novel feature set is capable of identifying plants which were not originally inoculated with the fungus at the start of the experiment but which subsequently developed disease through natural transmission.

## Introduction

Automatic detection of disease and water stress in plants and canopies is a developing area for the modern day horticulture and agriculture industry. The development of sophisticated instruments and fast computational techniques have paved the way for real-time scanning and automatic detection of anomalies in a crop [1–3]. In a recent study, it has been shown that image analysis can be used to provide a consistent, accurate and reliable method to estimate disease severity [4]. Multi-modal imaging has been used by researchers in the past for determining the quality of crop. Among various imaging techniques, thermal imaging has been shown to be a powerful technique for detection of diseased regions in plants [5]. One of the major problems associated with thermal imaging in plants is temperature variation due to canopy architecture, leaf angles, sunlit and shaded regions, environmental conditions and the depth (distance) of plant regions from the camera [6]. In this paper, we aim to combine information from stereo visible light images with thermal images to overcome these problems and present a method for automatic detection of disease in plants using machine learning techniques.

It is widely known that the thermal profile or the time interval between onset and visible appearance of disease varies depending on the type of disease and the plant. This paper is a step towards making automatic detection of disease possible regardless of disease or plant type. We present here a novel approach for automatic detection of diseased plants by including depth information to thermal and visible light image data. We study the effect of a fungus *Oidium neolycopersici* which causes powdery mildew in tomato plants and investigate the effect of combining stereo visible imaging with thermal imaging on our ability to detect the disease before appearance of visible symptoms. For depth estimation, we compare six different disparity estimation methods and propose a method to estimate smooth and accurate disparity maps with efficient computational cost. We propose two complimentary approaches to extract a novel feature set and show that it is capable of identifying plants poised to be affected by the fungus during the experiment.

## Related Work

Thermal imaging has good potential for early detection of plant disease, especially when the disease directly affects transpiration rate, as it can be shown that leaf temperature changes with the change in transpiration rate [7]. Early detection of disease is very important as prompt intervention (e.g. through the application of fungicides or other control measures) can control subsequent spread of disease which would result in reduced the quantity and quality of crop yield [7]. Naidu *et al*. [8] used discriminant analysis to identify virus infected grapevine (grapevine leafroll disease) using leaf reflectance spectra. The authors found specific differences in wavelength intervals in the green, near infrared and mid-infrared region and obtained a maximum accuracy of 81% in classification results. Chaerle *et al* [9] studied tobacco infected with tobacco mosaic virus (TMV) and found that sites of infection were 0.3–0.4°C warmer than the surrounding tissue approximately 8±1 hours before the initial appearance of the necrotic lesions. They also observed a correlation between leaf temperature and transpiration by thermography and steady-state porometry. Later, Chaerle *et al* [10] studied the use of thermal and chlorophyll fluorescence imaging in pre-symptomatic responses for diagnosis of different diseases and to predict plant growth. The authors concluded that conventional methods are time consuming and suitable for small number of plants, whereas imaging techniques can be used to screen large number of plants for biotic and abiotic stress and to predict the crop growth.

Oerke *et al* [11] studied the changes in metabolic processes and transpiration rate within cucumber leaves following infection by *Pseudoperonospora cubensis* (downy mildew) and showed that healthy and infected leaves can be discriminated even before symptoms appeared. The maximum temperature difference (MTD) was found to be related to the severity of infection and could be used for the discrimination of healthy leaves or those with downy mildew [12]. In another study, Oerke *et al* [13] investigated the effect of the fungus *Venturia inaequalis* on apple leaves and found MTD to be strongly correlated with the size of infection sites. Stoll *et al* [14] investigated the use of infrared thermography to study the attack of *Plasmopara viticola* in grape vine leaves under varying water status conditions while research on wheat canopies for detection of fungal diseases revealed that higher temperature was observed for ears (containing the grain) infected with *Fusarium* [7, 15].

In addition to colour and temperature information, we add depth information to our analysis in this study. Application of stereo vision in horticulture is not new and has been used for plant quality assessment and phenotyping previously. Ivanov *et al* [16] presented a feature-based matching approach for disparity estimation in stereo images of plants but it was not fully automatic. Andersen *et al* [17] and Biskup *et al* [18] used area correlation combined with simulated annealing to estimate depth. Song *et al* [19] presented a multi-resolution pyramid and Kalman filtering to update disparity results from one scale to the next. To increase the accuracy of 3D depth estimation, stereo vision has been combined by various researchers with Light Detection and Ranging (LIDAR) technology [20, 21]. Here, we use a stereo visible imaging setup for depth estimation to avoid any extra costs and computational burden being added to the setup by the addition of another imaging system.

## Materials and Methods

### 1 Image Acquisition

**Fig 1:**
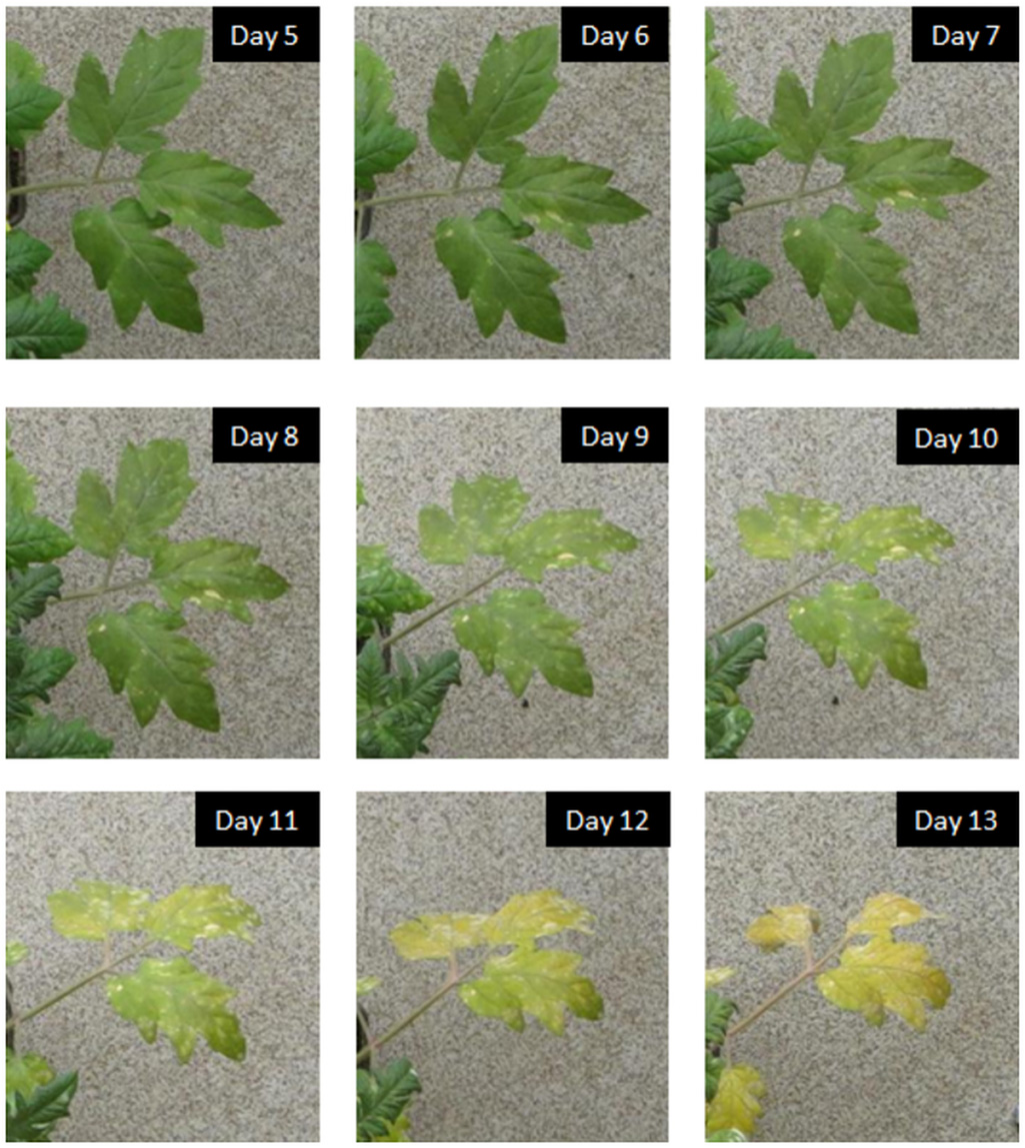
Progress of disease with time. The appearance of disease symptoms with time on leaves of a diseased plant.

An experimental setup was designed and developed at the Department of Computer Science, University of Warwick, UK, to simultaneously acquire visual and thermal images of diseased/healthy plants. The imaging setup consisted of two visible light imaging cameras (Canon Powershot S100), and a thermal imaging camera (Cedip Titanium). The experiment was carried out on 71 tomato plants (*cultivar Espero*) in a controlled environment at 20°C with thermal and stereo visible light images being collected for 14 consecutive days (day0 to day13). All the plants were watered at the same time of the day with approximately same amount of water to minimise the effect of plant water stress on the disease detection. Of these 71 plants, 54 plants were artificially inoculated on day0 with the fungus *Oidium neolycopersici* which causes powdery mildew disease, whereas the remaining 17 plants were not inoculated. Inoculation was carried out by spraying the tomato plants to run off with a spore suspension of *O. neolycopersici* at a concentration of 1 × 10^5^ spores *ml* ^−1^. The disease symptoms that developed consisted of white powdery lesions (first start to visibly appear after approximately 7 days) that expanded over time and eventually caused chlorosis and leaf die-back (Fig 1).

### 2 Pre-processing

**Fig 2:**
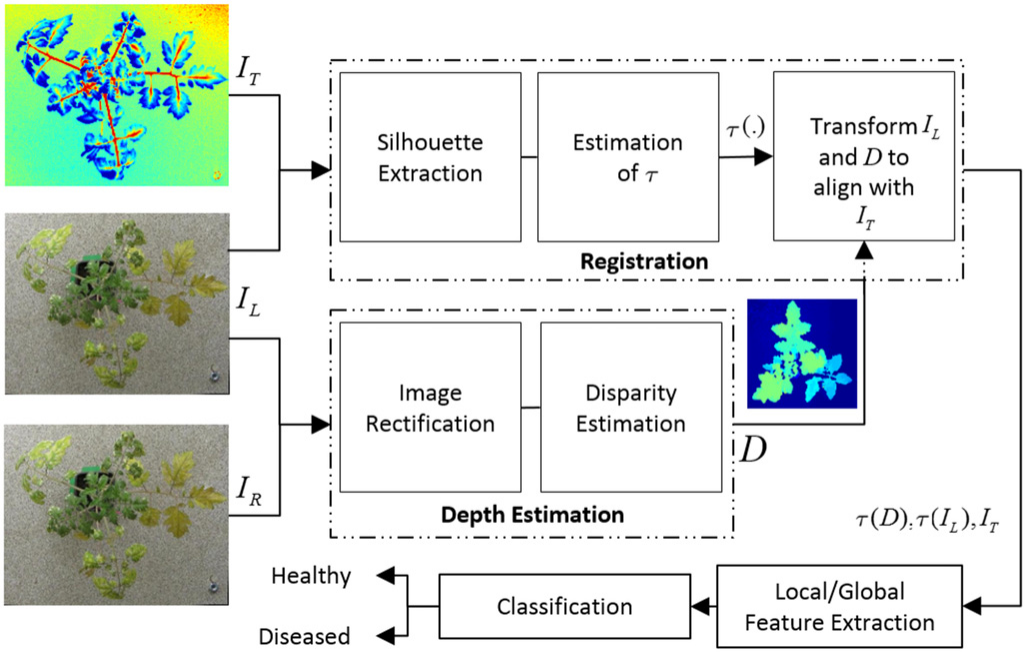
Block diagram for the proposed algorithm. Block diagram for the proposed algorithm for detection of diseased plants. The algorithm applies the transformation *τ* to align the colour image with the thermal image. *I*_*L*_ and *I*_*R*_ represent visible light images from left and right cameras, *I*_*T*_ represents the thermal image.

The block diagram of the proposed detection algorithm is shown in Fig 2. The detection algorithm consists of registration, depth estimation, feature extraction and classification. Before the extraction of features the pre-processing pipeline consists of registration and depth estimation as described in the remainder of this section.

#### 2.1 Image Registration

**Fig 3:**
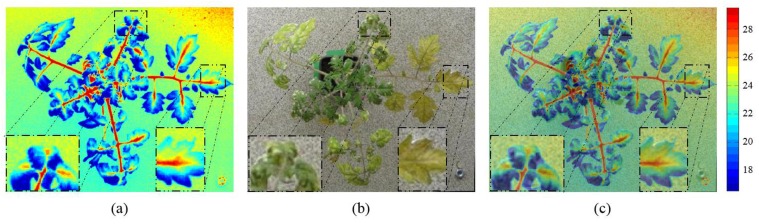
Registration of thermal and visible light images. Thermal image (a), visible light image (b), Overlay of thermal image on visible light image after registration (c). The blue shade represents lower temperature and the red shade represents high temperature values. It can be observed that high temperature stem regions in thermal image faithfully follow the stem regions in the visible light image. Similarly low temperature leaf regions overlap the leaf regions in the visible image.

The detection algorithm combines information from thermal, depth and visible light images of the plants and uses classification of features extracted from these images to detect a plant as healthy or diseased. As a pre-processing step before combined analysis, thermal and visible light images of plants must be aligned so that the same pixel locations in both the images correspond to the same physical locations in the plant. Registration of thermal and visible light images has been performed by various researchers for various applications [22, 23]. In this paper, we use the registration algorithm specifically designed by our group for registration of diseased plants [24]. Briefly, the algorithm first extracts the plant silhouette using a novel multi-scale silhouette extraction algorithm based on stationary wavelet transform. Using the extracted silhouettes, the algorithm then applies a global + local registration approach to estimate the transformation *τ* required to register salient features in pairs of thermal and visible light images. An overlay of thermal image on the corresponding visible light image is shown in Fig 3 (c) after registration. The blue shade represents lower temperature and the red shade represents high temperature values. It can be observed that high temperature stem regions in thermal image faithfully follow the stem regions in the visible light image. Similarly, low temperature leaf regions overlap the leaf regions in the visible image. The reader is referred to [24] for more details of the registration algorithm.

#### 2.2 Depth Estimation

To add depth information to the set of features which can be collected from registered thermal and visible light images, we use disparity between the stereo image pair. For a stereo vision setup, depth (*Z*) can be related to disparity (*d*) by *d* = *fB*/*Z*, where *f* is focal length of the lens of the camera and *B* is the baseline which can be defined as the distance between the centres of left and right camera lenses. In this paper, we propose a disparity estimation method for estimation of smooth and accurate disparity maps and compare the results with five state of the art existing methods. We selected these five method(s) for our study based on three criteria: 1) they represent major disparity estimation schemes; 2) these methods have been used in the past for comparison studies [25]; and 3) they produce acceptable results on the plant images. The goal is to develop a robust method which produces stable disparity maps in the presence of colour variation and background noise. We compare the following six methods in this paper:

1. Block-based Stereo Matching (BSM) [26]
2. Multi-Resolution Stereo Matching (MRSM) [19]
3. Graph-cut based Stereo Matching (GCM) [27]
4. Non-local Cost Aggregation (NCA) [28]
5. Semi-Global Matching (SGM) [29]
6. The proposed Multi-Resolution Semi-Global Matching (MRSGM)

**Fig 4:**
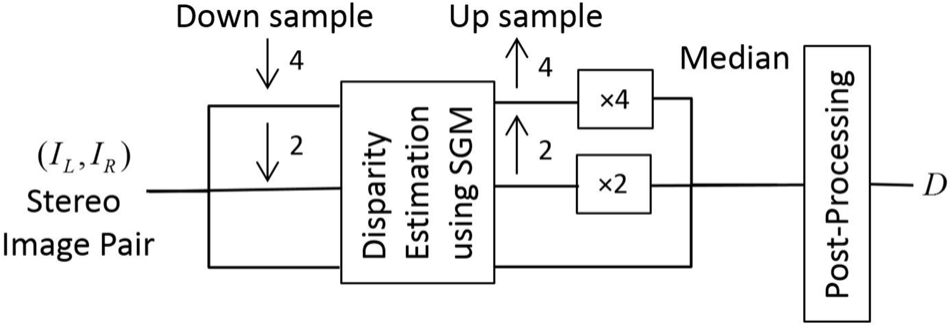
Disparity estimation. Overview of the proposed multi-resolution semi-global matching approach, where ↓ *n* and ↑ *n* denote down sampling and up sampling of the image by a factor of *n*.

Further detail about the first five disparity estimation methods can be found in the appendix. The proposed multi-resolution semi-global matching (MRSGM) method is based on SGM [29]. An overview of the proposed approach is shown in Fig 4. We first calculate the disparity at three consecutive dyadic resolutions and then take the median of the disparity estimated at these three resolutions. For disparity estimation, we use SGM with block based Birchfield and Tomasi (BT) cost function [30] as the matching cost instead of pixel-wise matching. After disparity estimation, we post-process the result to create a smooth and accurate disparity map which is robust to the background noise and variation in our data set using the colour information as proposed in [31]. We use bilateral filtering [32] as a post-processing method to improve the disparity map. The underlying assumption is that the colour discontinuity is a strong indicator of depth discontinuity. If *D* denotes the disparity map and *I* denotes the reference image, then for a pixel *p* = {*x*, *y*}, let us assume *d*_*p*_ = {*D*(*x* − 1, *y*), *D*(*x*, *y* − 1), *D*(*x* + 1, *y*), *D*(*x*, *y* + 1)}, *u*_*p*_ = {*x* − *r*,…, *x* + *r*}, *v*_*p*_ = {*y* − *r*,…, *y* + *r*}, where *r* is the radius of the bilateral filter. We can update the disparity map *D* using the following equation

$$\begin{array}{c} D\left(x,y\right)=\underset{d\in d_{p}}{\arg\min}\;\frac{\sum_{u\in u_{p}}\sum_{v\in v_{p}}\text{W}(u,v)\text{C}(u,v,d)}{\sum_{u\in u_{p}}\text{W}(u,v)} \end{array}$$ (1)

where

$$\begin{array}{c} \text{W}\left(u,v\right)=\exp(-\frac{1}{2}{\left(\frac{\left||I(x,y),I(u,v)|\right|}{\sigma_{r}}\right)}^{2}).\exp(-\frac{1}{2}{\left(\frac{\left||(x,y),(u,v)|\right|}{r}\right)}^{2}) \end{array}$$

$$\begin{array}{c} \text{C}(u,v,d)=\min(\gamma ℒ,|D(u,v)-d|) \end{array}$$

where ‖.‖ is *l*_2_-norm, *γ* is a constant and was chosen to be 0.2 [31], and 𝓛 is the total number of disparities. The remaining parameters *σ*_*r*_ and *r* can be used to control the smoothness of the updated disparity map.

### 3 Detection of Diseased Plants

**Fig 5:**
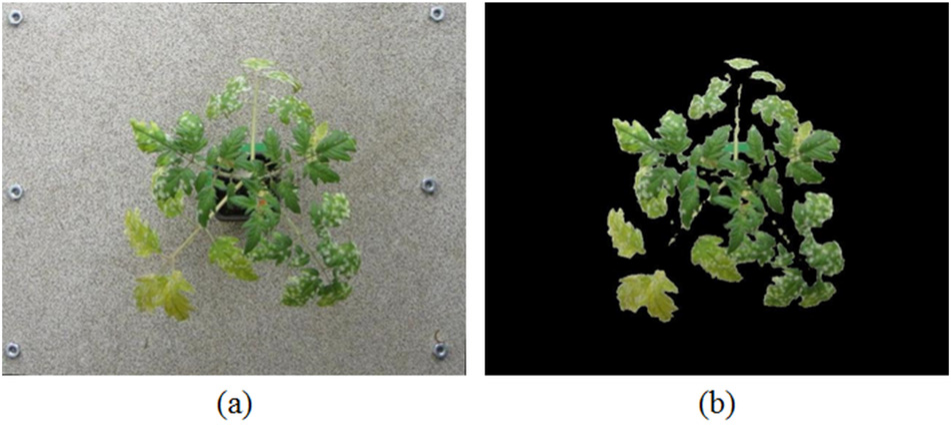
Background Removal. Colour image registered with thermal image (a); Colour image obtained after the background removal (b).

In this section, we combine depth, temperature and colour information from thermal and stereo visible light images. The transformation(s) estimated in Section 2.1 were used to align all the three images (thermal, colour and disparity) so that the same pixel location in all the three images corresponds approximately to the same physical point in the plant [24]. After registration, we remove the background to obtain an image which contains only plant regions. To remove the background, we train an Support Vector Machine (SVM) classifier with a linear kernel using the RGB pixel values by selecting small patches from foreground and background regions and classify each pixel into background/plant pixel. The result of extracting the plant region using our method on an image is shown in Fig 5 (b).

We present two complimentary feature extraction approaches, i.e. local and global, for extraction of useful information from the image data of plants. The extracted features are fed into a classifier and the classifier decides based on the input information whether the plant should be classified as healthy or diseased. The local feature extraction approach extracts the features from a selected set of pixels, whereas global feature extraction extracts the features from all the plant pixels. Further details about the feature extraction approaches is given in the remainder of this section.

#### 3.1 Local Feature Extraction

The local feature extraction is a two-step approach which directly uses colour, depth and temperature values to first identify potential diseased areas and then it extracts information from these areas (pixels) to be used for classification.

##### 3.1.1 Identification of Potential Diseased Areas

**Fig 6:**
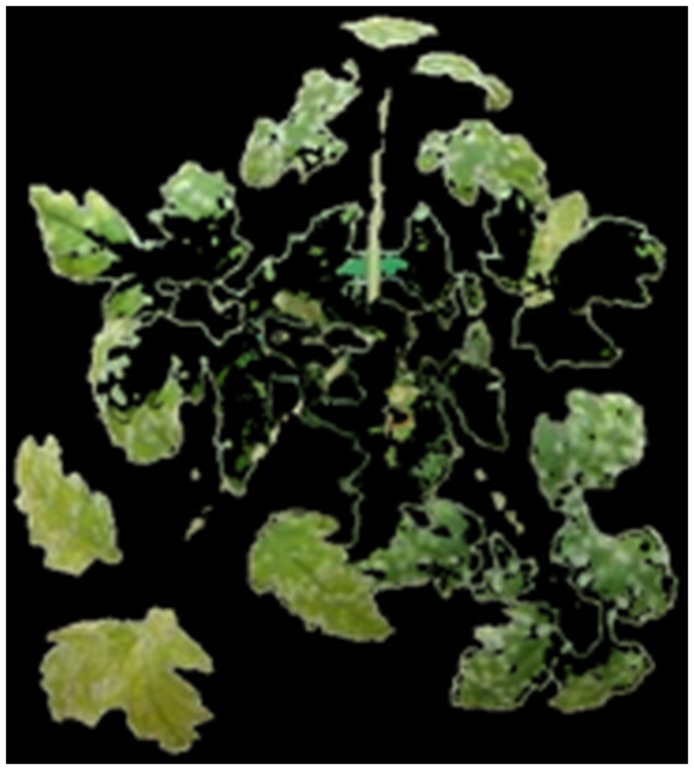
Identification of potential diseased areas. Identification of diseased areas in Fig 5 (b) by classification of feature vector $\vec{V}$ at each pixel.

For the identification of potential diseased areas, we convert the colour space of the RGB image in Fig 5 (b) to Lab. Similarly, we change the RGB colour space of the colour image to CMYK where C and Y channels correspond to the strength of cyan and yellow colours in the image, both carry the green colour. Two different colour spaces were used as they carry different type of information. In the Lab colour space, *a* and *b* carry all the colour information. In the CMYK colour model, C and Y are generated by combination of ‘green and blue’ and ‘green and red’ colour respectively. In addition, from the electromagnetic spectrum it can be analysed that C and Y carry information from the wavelengths which are very close to green colour thus broadening our search range while keeping the information as close to green colour as possible. Lab colour space model does not provide the flexibility provided by C and Y channels to analyse nearly green areas separately. We directly use the pixel values corresponding to *a* and *b* channels from Lab colour space and C & Y channels from CMYK colour space. For depth and temperature information, we directly use pixel values in disparity map *D* and thermal intensity map *T*, respectively. Therefore, our classifier uses a six dimensional feature vector $\vec{V}$ consisting of *a*, *b*, *C*, *Y*, *D* & *T* values at each pixel location. We train the SVM classifier kernel using small patches from healthy and diseased regions, to identify diseased pixels in an image using the feature vector $\vec{V}$, the result of diseased pixel identification for the image in Fig 5 (b) is shown in Fig 6.

##### 3.1.2 Selection of Features from the Potential Diseased Areas

**Fig 7:**
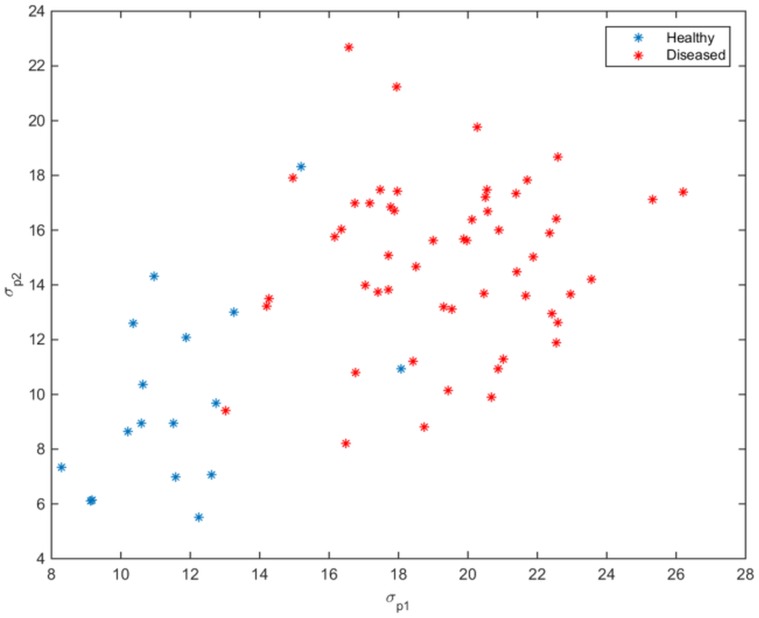
Classification using local feature set. Scatterplot of the standard deviation (*σ*_*p*1_ & *σ*_*p*2_) of data, corresponding to potentially diseased pixels for healthy and diseased plants (day13), along the 1^*st*^ and 2^*nd*^ principal components respectively.

The healthy plants are expected to provide smooth profile in thermal, colour and depth images compared to diseased plants. This prior knowledge leads us to an assumption that the healthy plants carry less variation in the aforementioned feature measurements whereas the diseased plants carry large variation in the same measurements. If our assumption is true, we must be able to detect diseased plants using temperature, colour and depth information. It is possible that some pixels in healthy plants can be erroneously classified as diseased pixels as a result of the first step (Section 3.1.1). According to our assumption, if a region in a healthy plant is incorrectly classified as diseased, it will have less variation whereas a correctly classified diseased region will have high variation. To test our hypothesis, we placed all the feature vectors corresponding to the diseased pixels in Fig 6 in a matrix **V** and performed the principal component analysis (PCA) on **V**. We computed the standard deviation of data along the first and second principal components as *σ*_*p*1_ & *σ*_*p*2_ respectively. The smaller values of *σ*_*p*1_ & *σ*_*p*2_ in Fig 7 for healthy plants validates our assumption that there is low variation in data for healthy plants compared to diseased plants, therefore we can detect diseased plants by classifying this information.

#### 3.2 Global Feature Extraction

In this approach, instead of computing features at each pixel location or a specific part of the plant, we directly compute features from all the pixels which belong to the plant, as shown in Fig 5 (b). We experimented with 45 different features, which were chosen based on the work done by researchers in the past [1, 7]. We found the following list of features to be most discriminating according to the *p*-values computed using analysis of variance (ANOVA) for different days after inoculation as described in Table 1: *μ*_*C*_, *μ*_*Y*_, *σ*_*sca*_, *σ*_*scL*_ and *σ*_*D*_. In this table, *μ*_*C*_ and *μ*_*Y*_ are the mean values of cyan and yellow colour in CMYK, as both carry information about the green colour. Yellow is very important as the leaf infected with powdery mildew turns yellow after showing white lesions. Therefore, the presence of yellow colour can be directly translated to disease. The next two features, i.e. *σ*_*scL*_ and *σ*_*sca*_, are standard deviation of temperature values scaled by Luminance and *a* channels. Luminance is important to get information about the light intensity whereas lower values of *a* carry information about the greenness of the pixel. The features denoted by *σ*_*scL*_ and *σ*_*sca*_ were also found to be very useful features in a previous study aimed at automatic detection of water deficient regions in a spinach canopy [1]. The feature *σ*_*D*_ carry depth information (in terms of disparity), the standard deviation of disparity must be higher in the diseased plants because of the irregular leaves, whereas in healthy plants it should be low because of smoother leaves.

**Table 1:** Extraction of global feature set. *p*-values of the separation power of selected feature set for day 5 to day 13 after inoculation computed using ANOVA.

Day	*μ*_*C*_	*μ*_*Y*_	*σ*_*sca*_	*σ*_*scL*_	*σ*_*D*_
5	0.75	0.67	4.17 × 10^−06^	0.59	0.02
6	0.30	0.46	1.41 × 10^−06^	0.81	5.33 × 10^−03^
7	0.11	0.18	9.07 × 10^−06^	0.33	6.85 × 10^−04^
8	0.01	0.08	2.18 × 10^−05^	0.10	1.30 × 10^−04^
9	4.39 × 10^−04^	9.40 × 10^−03^	3.78 × 10^−05^	4.54 × 10^−04^	1.14 × 10^−07^
10	3.66 × 10^−05^	8.54 × 10^−05^	1.37 × 10^−05^	4.07 × 10^−05^	9.71 × 10^−09^
11	3.77 × 10^−05^	2.66 × 10^−06^	1.94 × 10^−07^	8.37 × 10^−07^	7.49 × 10^−11^
12	5.27 × 10^−06^	4.09 × 10^−09^	3.46 × 10^−06^	3.12 × 10^−10^	6.52 × 10^−13^
13	1.35 × 10^−06^	1.23 × 10^−10^	5.47 × 10^−05^	5.80 × 10^−11^	1.48 × 10^−12^

## Results and Discussion

### 4.1 Depth Estimation

All the algorithms and results presented in this section were generated using a machine running Windows 7 on an Intel Core i3-2120 (3.3 GHz) CPU with 3GB RAM (665 MHz). The code for MRSM (provided by the author) was implemented in MATLAB 2013a, whereas the C/C++ implementation of GCM and NCA were downloaded from the author's websites. We used OpenCV library to implement SGM in C++ for our experiments. The BSM and MRSGM were partially implemented in C++ and partially in MATLAB 2013a, where the post processing algorithm in MRSGM uses C++ implementation by [31].

**Fig 8:**
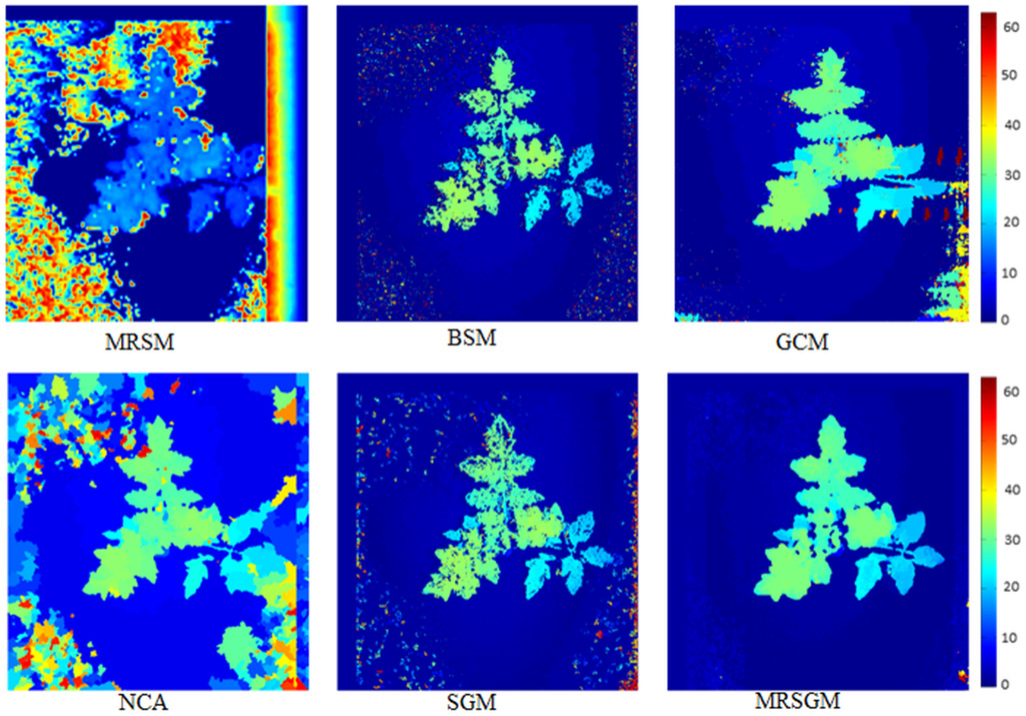
Comparison of disparity estimation results. Disparity estimation results of methods in Section 2.2 on the stereo plant image. The colour bar on the right shows disparity values in pixels.

To validate our method, we have compared the results of the proposed MRSGM with the remaining five methods in the S1 Appendix. We have shown that our method not only produces decent results on standard test datasets but is also computationally efficient compared to other methods. Fig 8 compares results of all the six methods on our dataset. It shows that MRSM performed poorly on the plant images and was found to be very sensitive to the background noisy pattern in the image. From the results on test images from Middlebury dataset (S1 Appendix), we know that GCM and NCA produce accurate disparity maps but in the case of plant images these two algorithms were found to be highly sensitive to the noise content in the image. GCM is slow and produces artifacts along the scan lines on the plant images. The NCA algorithm divides the image into regions and assumes a constant disparity throughout this region, this approach sometimes produces false disparity maps specially in diseased parts of the plant. The false disparity maps appear as artifacts which can be observed in NCA result. BSM and SGM results were found to be less sensitive to background noise but the disparity map produced by these methods were not smooth and showed small peaks/patches around some pixels which were inconsistent with the neighbouring disparity. When compared to all the other methods, MRSGM produced robust disparity maps which were found to be less sensitive to the noise. Although GCM and NCA performed well on the test datasets, our plant images with relatively more background noise than the Middlebury images proved to be quite challenging for these algorithms. In addition, GCM and NCA were found to be much slower (Figure D in S1 Appendix) compared to the proposed MRSGM which was found to be not only less sensitive to the noisy pattern but also produced smooth and accurate disparity maps. In the following section, we present and discuss the results of detection of diseased plants with and without disparity features.

### 4.2 Detection Results

In this section, we present results of diseased plant detection using classification of local and global feature sets.

#### 4.2.1 Classification using Local Feature Set

**Fig 9:**
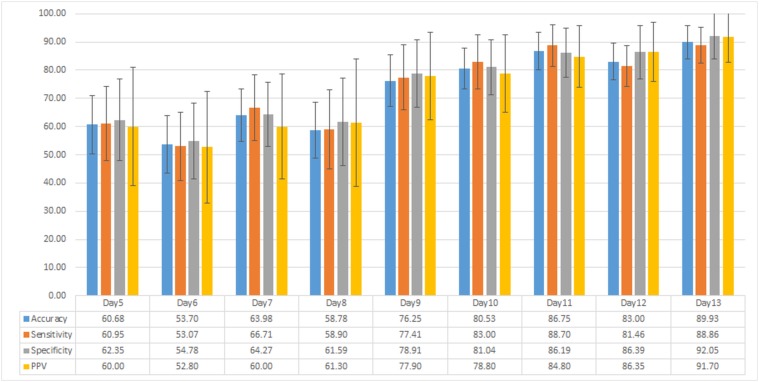
Classification results using local feature set. Average accuracy, sensitivity, specificity and positive predictive value (PPV) results of diseased plant detection using classification of local feature set.

From the total of 71 plants, 54 plants were diseased and 17 plants were healthy (not inoculated with the fungus). To test the strength of our local features, we used SVM classifier. We ran 200 cross-validation trials and tested the classifier using random pairs of training and testing data. In each trial, we randomly picked 17 out of 54 diseased plants for classification purpose. Once the number of diseased and healthy plants was equal, we randomly picked 7 out of 17 healthy and diseased plants each for training purpose and the remaining 10 for testing the classifier. The detection results of the proposed classifier using local feature set for 200 trials in terms of average accuracy, sensitivity, specificity and positive predictive value (PPV) are shown in Fig 9. The disease starts to appear 7 days after inoculation and, therefore, we concentrate on classification results for day5 to day13 after inoculation. Fig 9 indicates that we can achieve an average accuracy of more than 75%, 9 days after inoculation. The highest average accuracy achieved in this case is on day13, i.e. 89.93%, which is reasonably high. However, as the disease starts to appear 7 days after inoculation detecting the disease after day9 is not very beneficial at the commercial scale as it might spread across the crop. In the next section, we show that we can improve the accuracy of detection of diseased plants using the global feature set.

#### 4.2.2 Classification using Global Feature Set

**Fig 10:**
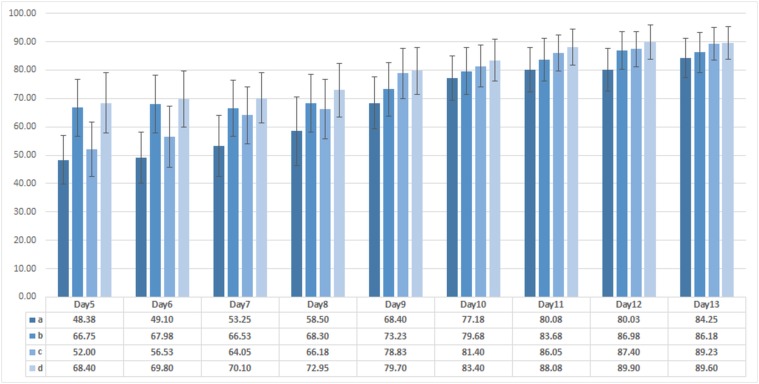
Comparison of classification results using different combination of features. Average accuracy of detection algorithm using different combination of global features. a, b, c & d show diseased plant detection results using classification of colour only, colour + thermal, colour + depth and colour + thermal + depth features respectively. Combining colour information with thermal or depth slightly increases the accuracy of the classifier, however combining colour information with thermal and depth improves the accuracy to approximately 70% on day5.

**Fig 11:**
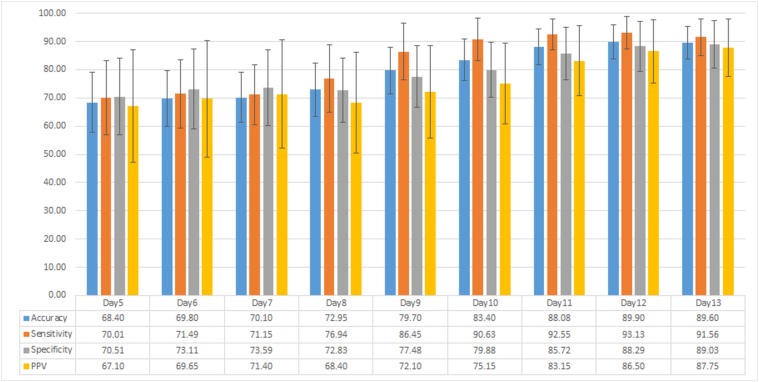
Classification results using global feature set. Average accuracy, sensitivity, specificity and positive predictive value (PPV) results of diseased plant detection using classification of global feature set.

We use the same classifier and the evaluation procedure as in Section 4.2.1, i.e. we use SVM and 7 images from each group for training and 10 images for testing for 200 cross-validation trials. For the purpose of comparative analysis, we divide our analysis to colour only (*μ*_*C*_, *μ*_*Y*_), colour + thermal (*μ*_*C*_, *μ*_*Y*_, *σ*_*sca*_, *σ*_*scL*_), colour + depth (*μ*_*C*_, *μ*_*Y*_, *σ*_*D*_, *μ*_*D*_), and colour + thermal + depth (*μ*_*C*_, *μ*_*Y*_, *σ*_*sca*_, *σ*_*scL*_, *σ*_*D*_), features to test how these different sets of features compare in terms of their ability to differentiate between healthy and diseased plants. From Fig 10, we can see that if we use only colour information we achieve accuracy of over 75% only after day10 of inoculation. We can increase this accuracy by combining colour information with thermal or depth, over 70% is achieved on and after day9, which is an improvement but again is not very beneficial to use at commercial scale. Combining the features from colour, thermal and disparity images increases the accuracy of our classifier to be approximately 70% on day5 and day6. Average accuracy of colour + thermal + depth feature set using global feature set in Fig 10 clearly outperforms results in Fig 9 of local feature set. Fig 11 show average accuracy, sensitivity, specificity and PPV values of disease detection using classification of global feature set.

#### 4.2.3 Investigation into Misclassification

**Fig 12:**
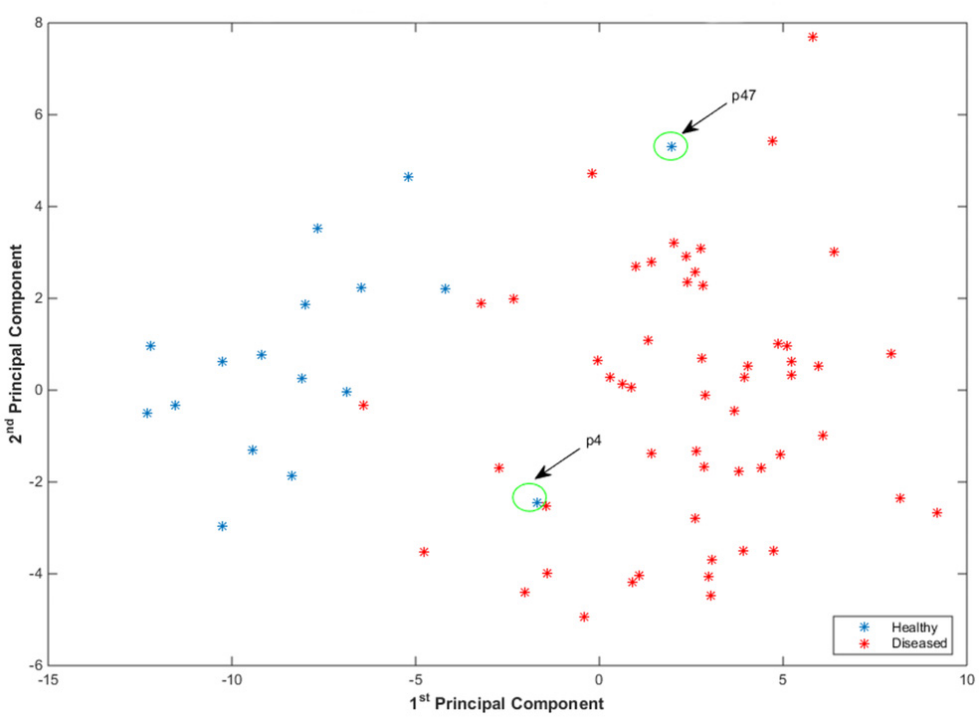
Identification of plants which captured the disease through natural transmission. Projection of features on 1^*st*^ and 2^*nd*^ principal component after performing PCA. The projection shows feature values corresponding to some of the plants which were not inoculated with any disease, occur in disease regions. A couple of these plants are marked as p4 & p47 and are shown in Fig 13.

**Fig 13:**
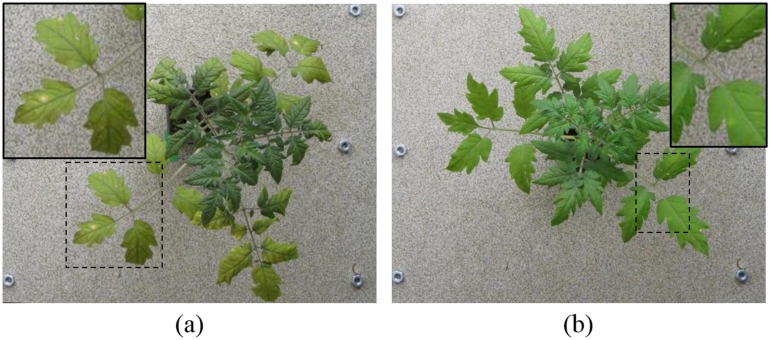
Examples which captured the disease through natural transmission. The plants (a) p47 & (b) p4 shown for illustrative purpose, the plants were not inoculated with any disease but later showed symptoms of the disease. These plants were successfully captured by our novel feature set.

**Fig 14:**
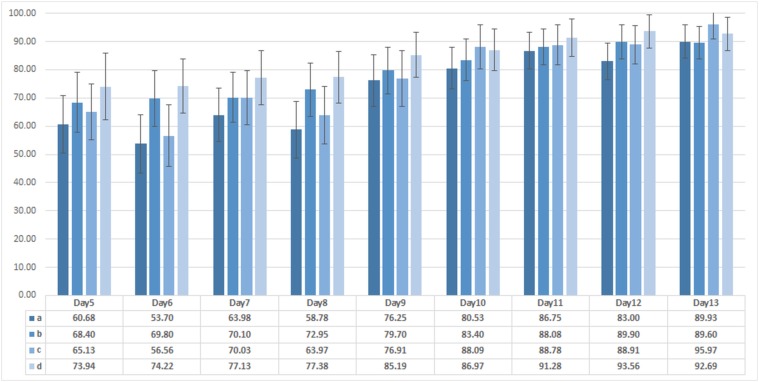
Comparison of classification results by marking the plants as diseased which captured the disease through natural transmission. Average accuracy results of diseased plant detection using classification of a) local feature set, b) global feature set, c) local feature set with p4 & p47 marked as diseased and d) global feature set with p4 & p47 marked as diseased.

To investigate into misclassification errors, we performed PCA after combining the local and global features. Consider the projection of the features on the 1^*st*^ and 2^*nd*^ principal components, as shown in Fig 12. The projection shows feature values corresponding to some of the healthy plants in diseased regions. A couple of these plants are marked as p4 & p47 and are shown in Fig 13. These plants were initially not inoculated with disease but showed symptoms of disease subsequently during the experiment due to natural transmission. It is important to mention here that both inoculated and non-inoculated plants were kept in the same location so that they were subject to identical environmental conditions. To prevent cross-infection, uninoculated plants would need to be in a separate location where conditions would not be the same, leading to another source of variability. We redesigned our experiment where we marked the plants p4 & p47 as plants inoculated with the fungus. After 200 random cross-validation trials, we achieved an average accuracy of more than 90% on day13 using local or global feature sets as shown in Fig 14.

**Fig 15:**
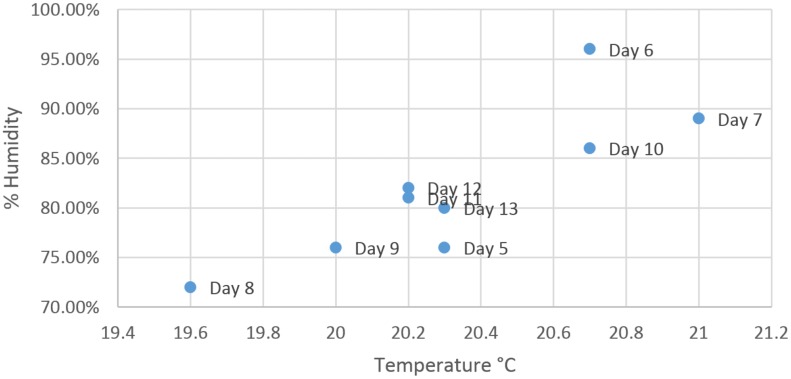
Temperature vs Humidity. Temperature vs Humidity plots as recorded from day5 to day13 of the experiment.

As mentioned before, the disease symptoms start to appear on day7 of inoculation and average accuracy of the detection algorithm is expected to increase with time after day7. However, the local feature set shows a decrease in the detection accuracy on day8 of inoculation (Fig 9). To investigate the decrease in average accuracy, the data from the experiment was carefully observed where it was found that on day8 of inoculation, the air temperature and humidity were recorded at the lowest levels due to practical limitations of the controlled environment as shown in Fig 15. The global feature set does not show this variable behaviour (Fig 11) and presents steady increase in accuracy with time. This is due to the fact that the global feature set compensates for changing environmental conditions by incorporating luminance and colour information into temperature data whereas the local feature set directly uses temperature values. The identification of naturally diseased plants among the non-inoculated plants and robustness of the proposed novel feature set to changing environmental conditions show the quality of our feature set and reliability of the proposed detection algorithm.

## Conclusions

Our results show that by combining information from thermal and stereo visible light images and using machine learning techniques, tomato plants infected with *O. neolycopersici* can be identified with high accuracy—more than 90%—significantly improving the use of remote images in the detection of disease onset. This improvement may be translated to other plant diseases and with further development of equipment may be used in a commercial setting. The proposed algorithm consisted of four parts: image registration, depth estimation, feature extraction and classification. Our novel multi-resolution method for depth estimation was shown to be computationally fast and less sensitive to noise while simultaneously producing smooth disparity maps.

In a local setting, we combined information to first detect potential disease areas in a plant and then we classified the plant into diseased or healthy plant based on the information collected from the potential disease areas. The global approach, on the other hand, aimed to detect disease from the whole plant. The average percentage agreement between the classification results produced by local and global feature set was found to be approximately 90% on day13. However, the disease detection results were found to be less stable and more sensitive to the environmental conditions in the local setting. The global feature set, on the other hand, shows a steady increase in detection accuracy with days compared to local feature set.

Our experimental results demonstrated that by combining colour information with thermal and depth information, the disease can be detected more accurately. It is important to mention here that the disease lesions *start* to appear on day7 but are *usually* not visible to the naked eye in most cases on this day. Therefore, colour features alone do not provide good result on detection, during early days, as the disease lesions are not clearly visible in most cases. But as the disease progresses, the disease lesions become more visible with every passing day and the difference between detection results provided by colour features alone and colour features combined with thermal and depth features reduces. Therefore, we added depth and thermal information to the feature set which provided higher accuracy compared to colour only features especially during early days. Although thermal information has been used in the past for disease and water stress detection, the addition of depth information for disease detection is novel.

The detection algorithm presented in this paper to identify individual plants infected with a disease can potentially be extended to whole canopy scanning in a commercial setting in future. However, the approaches presented here would need to be tested on a range of different plant diseases at a larger scale in order to assess the broad application of the techniques before they could be employed in a real world setting. Some of the features presented in our work, e.g. *σ*_*sca*_ and *σ*_*scL*_ also performed consistently well for drought detection in plants [1] in a previous work by our group. This consistency proves the potential of imaging techniques and the use of remote sensing in abiotic and biotic stress detection in plants. In addition, data from other imaging techniques such as spectral imaging and fluorescence imaging can be combined to improve accuracy. The use of these techniques for disease detection has great potential for temporal and spatial analysis of pathogen development. This would be useful practically for early disease detection to enable efficient and targeted use of pesticides, and also as a research tool to assess the efficacy of new control measures and to further understand the effect of different environmental factors on disease development. In the future, it would also be interesting to investigate if our technique could be used to classify different abiotic and biotic stresses such as water stress and disease.

## Supporting Information

S1 Appendix Comparison of Disparity Estimation Methods. (PDF)

S1 Source Code Source Code along with the results has been uploaded. (ZIP)

S1 Data Data for Day 5. (MAT)

S2 Data Data for Day 6. (MAT)

S3 Data Data for Day 7. (MAT)

S4 Data Data for Day 8. (MAT)

S5 Data Data for Day 9. (MAT)

S6 Data Data for Day 10. (MAT)

S7 Data Data for Day 11. (MAT)

S8 Data Data for Day 12. (MAT)

S9 Data Data for Day 13. (MAT)
