# Supplementary material for: Automatic Detection of Diseased Tomato Plants Using Thermal and Stereo Visible Light Images
Source: PLoS One. 2015 Apr 10;10(4):e0123262. doi: 10.1371/journal.pone.0123262 (PMC4393321; doi:10.1371/journal.pone.0123262)
Supplement: S1 Appendix — (PDF) [file pone.0123262.s001.pdf]

Appendix: Comparison of Disparity Estimation Methods

We compare the following six disparity estimation methods in this appendix

(1) Block-based Stereo Matching (BSM) [1]

(2) Multi-Resolution Stereo Matching (MRSM) [2]

(3) Graph-cut based Stereo Matching (GCM) [3]

(4) Non-local Cost Aggregation (NCA) [4]

(5) Semi-Global Matching (SGM) [5]

(6) The proposed Multi-Resolution Semi-Global Matching (MRSGM).

The first two methods in our list are based on block-based matching. The first method known as block-based stereo matching (BSM) [1], takes the Laplacian of Gaussian (LoG) transform of the stereo images and then uses absolute differences to find the matching blocks. A Multi-Resolution Stereo Matching (MRSM) was designed for surface modelling of plants in [2]. The algorithm first divides the image into overlapping blocks at each level of a multi-resolution pyramid and then uses a variation of the Birchfield and Tomasi (BT) cost function to match the corresponding blocks [6].

Graph-cut based Stereo Matching (GCM) [3] is a widely used disparity estimation method. This algorithm defines a global energy function and minimises the energy function using graph cuts [3,7–9]. The algorithm initially defines a unique disparity α and then iteratively searches for an α which minimises the energy function. The fourth method non-local cost aggregation (NCA) [4] uses the concept of bilateral filter by weighting the pixel intensity differences with intensity edges and provides a non-local solution by aggregating the cost on a tree structure derived from the stereo image pair.

Semi-Global Matching (SGM) [5] simplifies the energy minimisation problem by aggregating 1D *minimum* costs at each pixel from all directions. The cost $L_r(p, d)$ of pixel p at disparity d

along the direction *r* can be defined as

$$\begin{aligned} L_r(p,d) = {} & \mathrm{C}(p,d) + \min(L_r(p-r,d), L_r(p-r,d-1) + \mathrm{P}_1, \\ & L_r(p-r,d+1) + \mathrm{P}_1, \min_i L_r(p-r,i) + \mathrm{P}_2) \\ & - \min_k L_r(p-r,k) \end{aligned} \tag{A.1}$$

The first term on the right hand side in the above equation is the pixel-wise matching cost $\mathrm{C}(p,d)$. P_1 adds a small penalty for all pixels *q* in the neighbourhood *N* of pixel *p* for which disparity changes are small. P_2 adds large penalty for large changes in disparity in the neighbourhood of pixel *p*. The disparity *d* at pixel *p* can then be determined by minimising the cost $S(p,d)$

$$S(p,d) = \sum_r L_r(p,d) \tag{A.2}$$

Details about the MRSGM method are provided in the manuscript.

Comparative Results

Figure A shows the six images from Middlebury dataset used to compare all the disparity estimation methods used in our experiments. Images labelled ‘Aloe’, ‘Baby 1’, ‘Bull’, ‘Flower Pots’, ‘Rocks 1’ were taken from the Middlebury dataset whereas the ‘Plant’ image shows a sample plant image from our dataset. The ground truth disparity map for the first five images was also provided with the Middlebury data set. To measure the quality of our results, we compute two quality measures as suggested by [10]. If D_c represents the disparity map estimated by the algorithm being tested and D_{GT} represents the ground truth disparity then we define RMS and *B* (percentage of bad matching pixels) as follows,

$$RMS = \sqrt{\left(\frac{1}{N_d} \sum_{(x,y)} |\Upsilon(x,y)|^2 \right)} \tag{A.3}$$

$$B = \left(\frac{1}{N_d} \sum_{(x,y)} \mathrm{T}(\Upsilon(x,y), \delta_d) \right) \quad \text{(A.4)}$$

where $\Upsilon(x, y) = |D_c(x, y) - D_{GT}(x, y)|$, N_d is the total number of pixels and δ_d is the disparity error tolerance. $\mathrm{T}(\Upsilon, \delta_d) = 1$ if $\Upsilon > \delta_d$ else $\mathrm{T}(\Upsilon, \delta_d) = 0$. We chose the tolerance value δ_d to be 1 pixel for the results presented here.

For BSM, we chose 11×11 block size and for MRSM, we used 16×16 with 2 pyramid levels for our experiments. For GCM, NCA and SGM, we chose default parameters provided by the authors. Finally, we chose 5×5 block-based BT as cost function and r=3, $\sigma_r = 15$ for MRSGM. All the parameters specified above other than the default parameters were chosen on the basis of their good results on stereo images of diseased plants.

Consider the plots shown in Figure B to Figure D. The MRSM algorithm [2], aims to produce smooth disparity maps but inadvertently increases the error in an attempt to produce smooth disparity maps, resulting in large errors in RMS and *B* plots as compared to all the other algorithms. In Figure D we do not include MRSM as it was implemented in MATLAB and is expected to be slow compared to the algorithms implemented in C/C++. However, efficiency of the algorithm in terms of time is irrelevant if the RMS error and *B* are very high. All the other algorithms produce comparable results for RMS error and *B* as shown in Figure B and Figure C respectively.

Figure D compares computational efficiency of the algorithms. GCM was found to be roughly more than 100 times slower than MRSGM, and NCA was calculated to be at least 3.5 times slower than MRSGM on the plant images. SGM and BSM performed faster computation compared to MRSGM. GCM and NCA were implemented in C/C++ whereas MRSGM, SGM and BSM were partially implemented in MATLAB and partially in C/C++. These results lead to the conclusion that although GCM and NCA produce more accurate results, they are slow

compared to MRSGM, SGM and BSM.

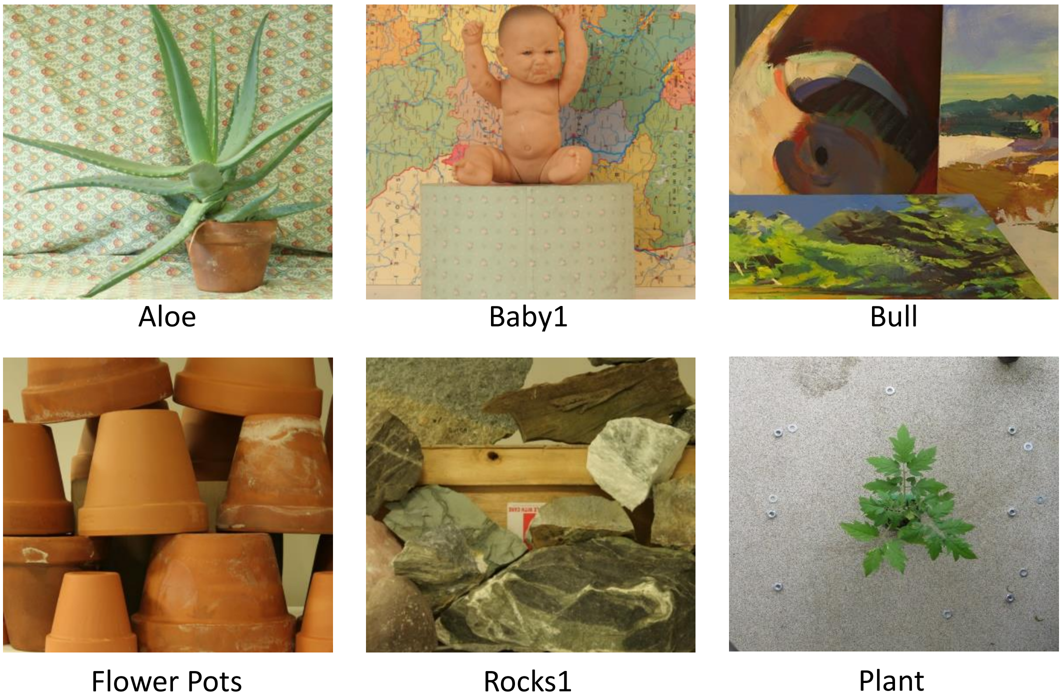

Figure A: ‘Aloe’, ‘Baby 1’, ‘Bull’, ‘Flower Pots’, ‘Rocks 1’ were taken from Middleburry dataset whereas the ‘Plant’ image shows a sample plant image from our data set.

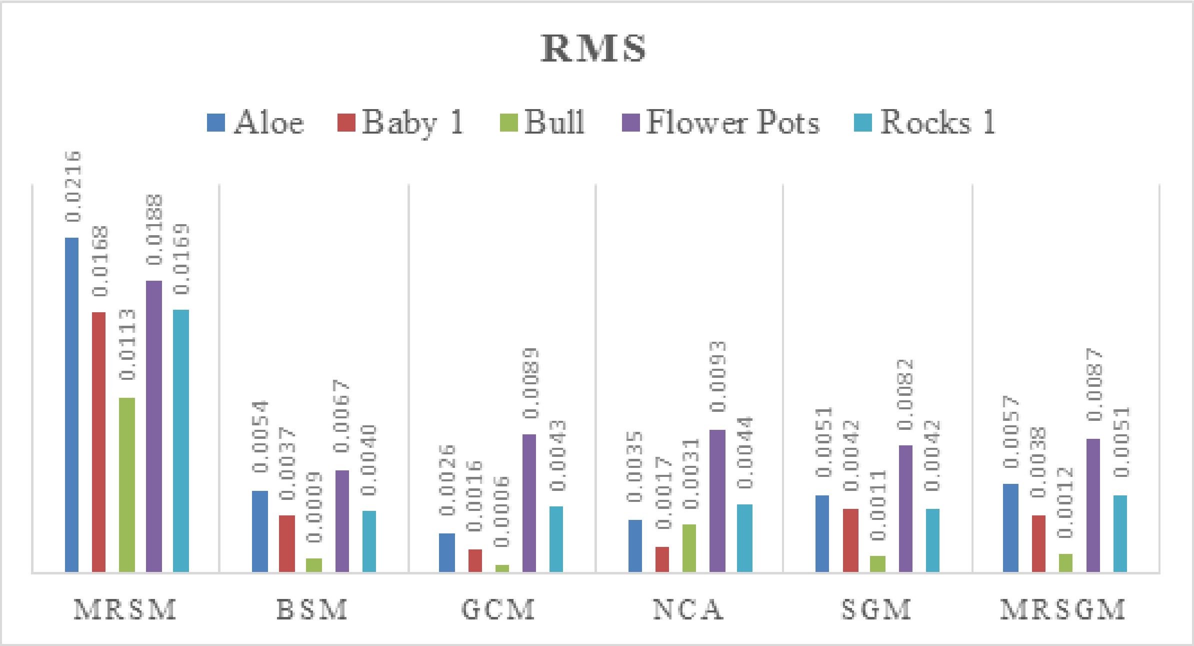

Figure B: RMS plots for five different images from Middleburry dataset shown in Figure A using the six disparity estimation methods.

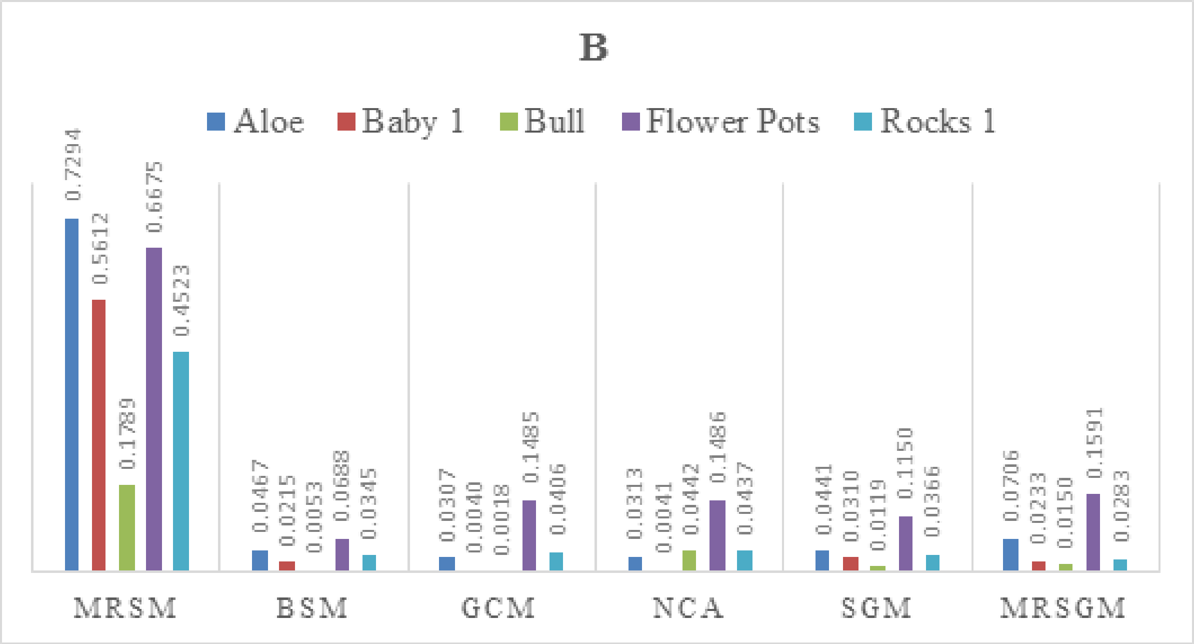

Figure C: *B* value plots for five different images from Middlebury dataset shown in Figure A using the six disparity estimation methods.

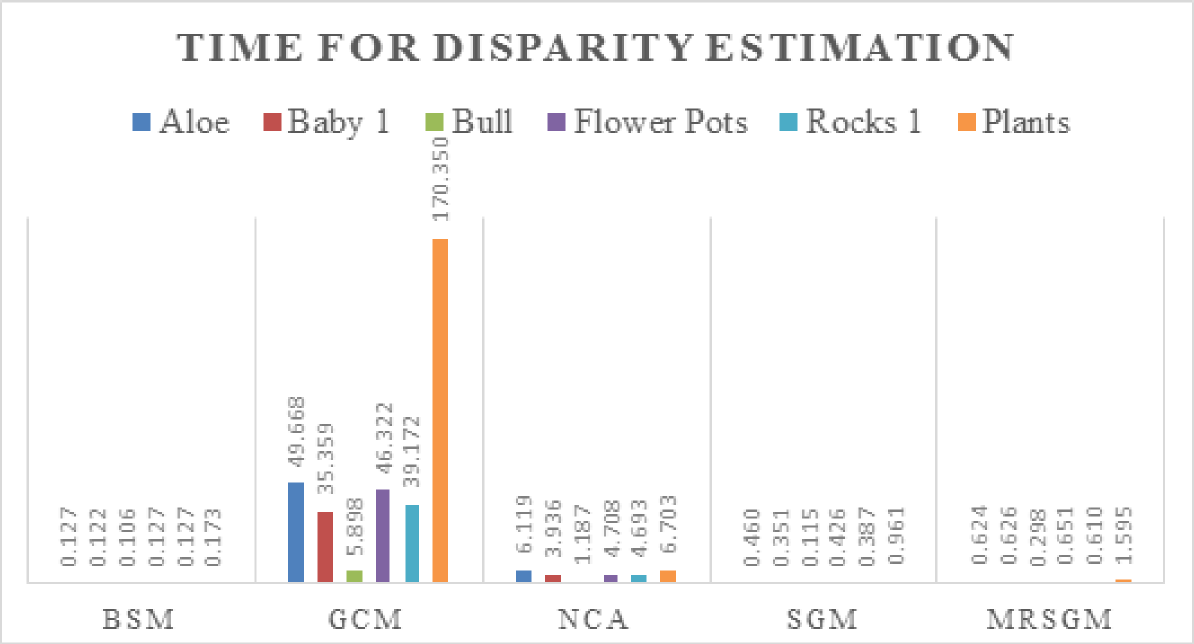

Figure D: Time taken (in seconds) for disparity estimation of images in Figure A using the six disparity estimation methods.

References

1. Konolige K (1998) Small vision systems: hardware and implementation. Robot Res Int. doi:10.1007/978-1-4471-1580-9_19.

2. Song Y, Wilson R, Edmondson R, Parsons N (2007) Surface Modelling of Plants from Stereo Images. Sixth International Conference on 3-D Digital Imaging and Modeling

(3DIM 2007). IEEE. pp. 312–319. doi:10.1109/3DIM.2007.55.

3. Kolmogorov V, Zabih R (2001) Computing visual correspondence with occlusions using graph cuts. Proc Ninth IEEE Int Conf Comput Vis 2: 508–515. doi:10.1109/ICCV.2001.937668.

4. Yang Q (2012) A non-local cost aggregation method for stereo matching. IEEE Conf Comput Vis Pattern Recognit: 1402–1409. doi:10.1109/CVPR.2012.6247827.

5. Hirschmüller H (2008) Stereo processing by semiglobal matching and mutual information. Pattern Anal Mach Intell IEEE Trans 30: 328–341. doi:10.1109/TPAMI.2007.1166.

6. Birchfield S, Tomasi C (1999) Depth discontinuities by pixel-to-pixel stereo. Int J Comput Vis 35: 269–293. doi:10.1023/A:1008160311296.

7. Boykov Y, Kolmogorov V (2004) An experimental comparison of min-cut/max-flow algorithms for energy minimization in vision. Pattern Anal Mach Intell IEEE Trans 26: 1124–1137. doi:10.1109/TPAMI.2004.60.

8. Kolmogorov V, Zabih R (2002) Multi-camera scene reconstruction via graph cuts. Comput Vision—ECCV 2002. doi:10.1007/3-540-47977-5_6.

9. Boykov Y, Veksler O, Zabih R (2001) Fast approximate energy minimization via graph cuts. IEEE Trans Pattern Anal Mach Intell 23: 1222–1239. doi:10.1109/34.969114.

10. Scharstein D, Szeliski R (2002) A taxonomy and evaluation of dense two-frame stereo correspondence algorithms. Int J Comput Vis 47: 7–42. doi:10.1023/A:1014573219977.
