# Supplementary figures and images for: Automatic Detection of Diseased Tomato Plants Using Thermal and Stereo Visible Light Images
Source: PLoS One. 2015 Apr 10;10(4):e0123262. doi: 10.1371/journal.pone.0123262 (PMC4393321; doi:10.1371/journal.pone.0123262)

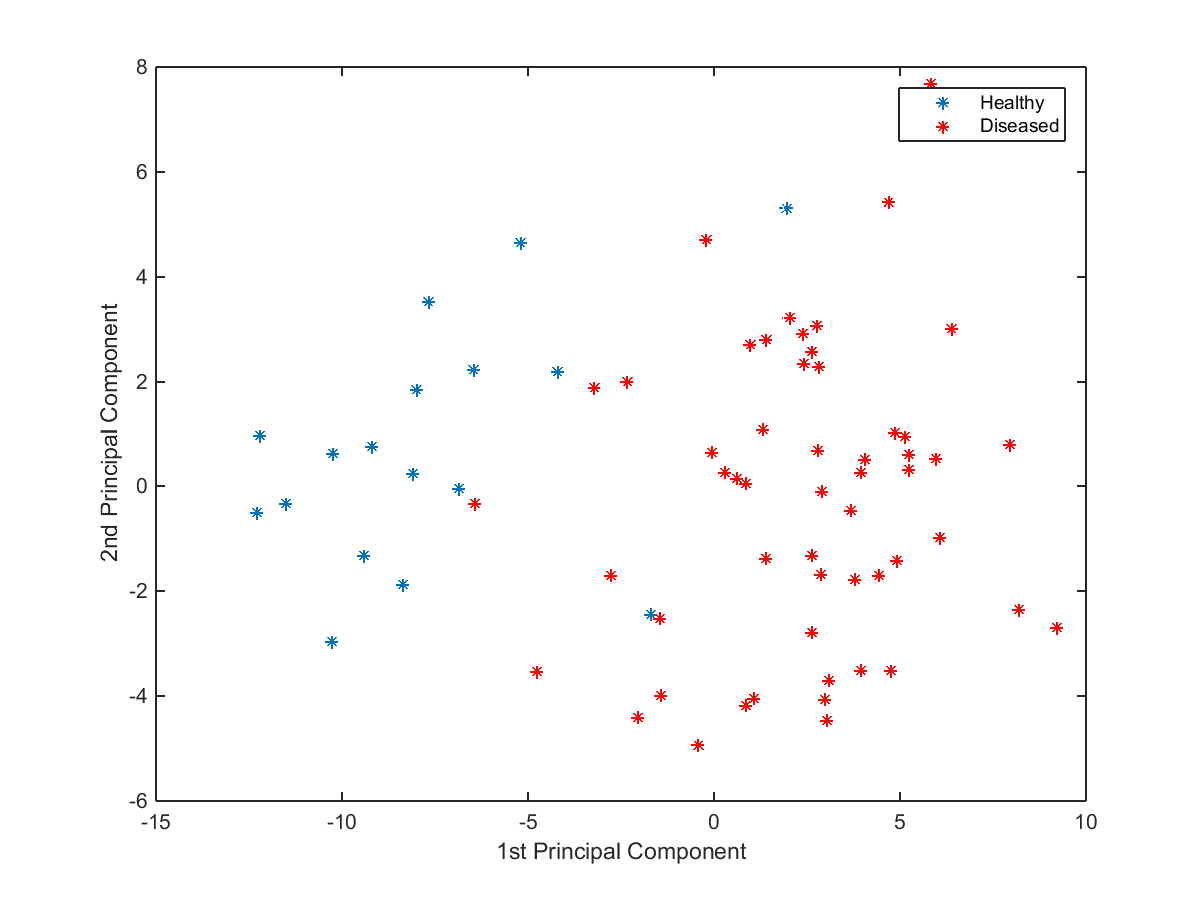

Supplement: S1 Source Code — (ZIP) [file pone.0123262.s002.zip › Results/Figure_12.png]

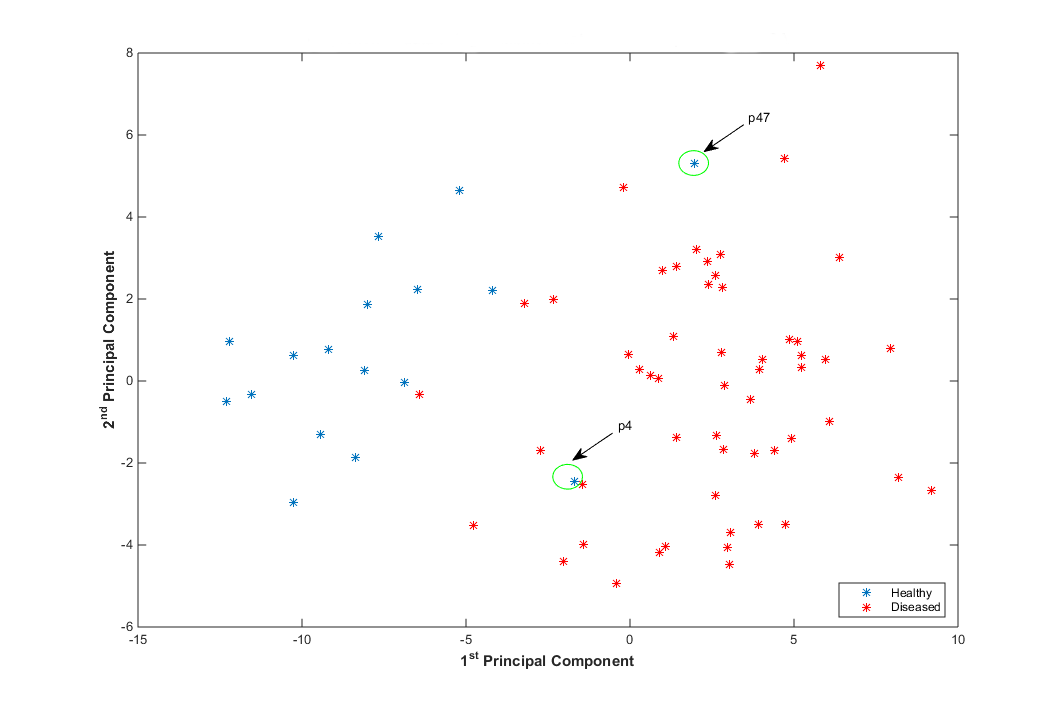

Supplement: S1 Source Code — (ZIP) [file pone.0123262.s002.zip › Results/Figure_12_1.png]

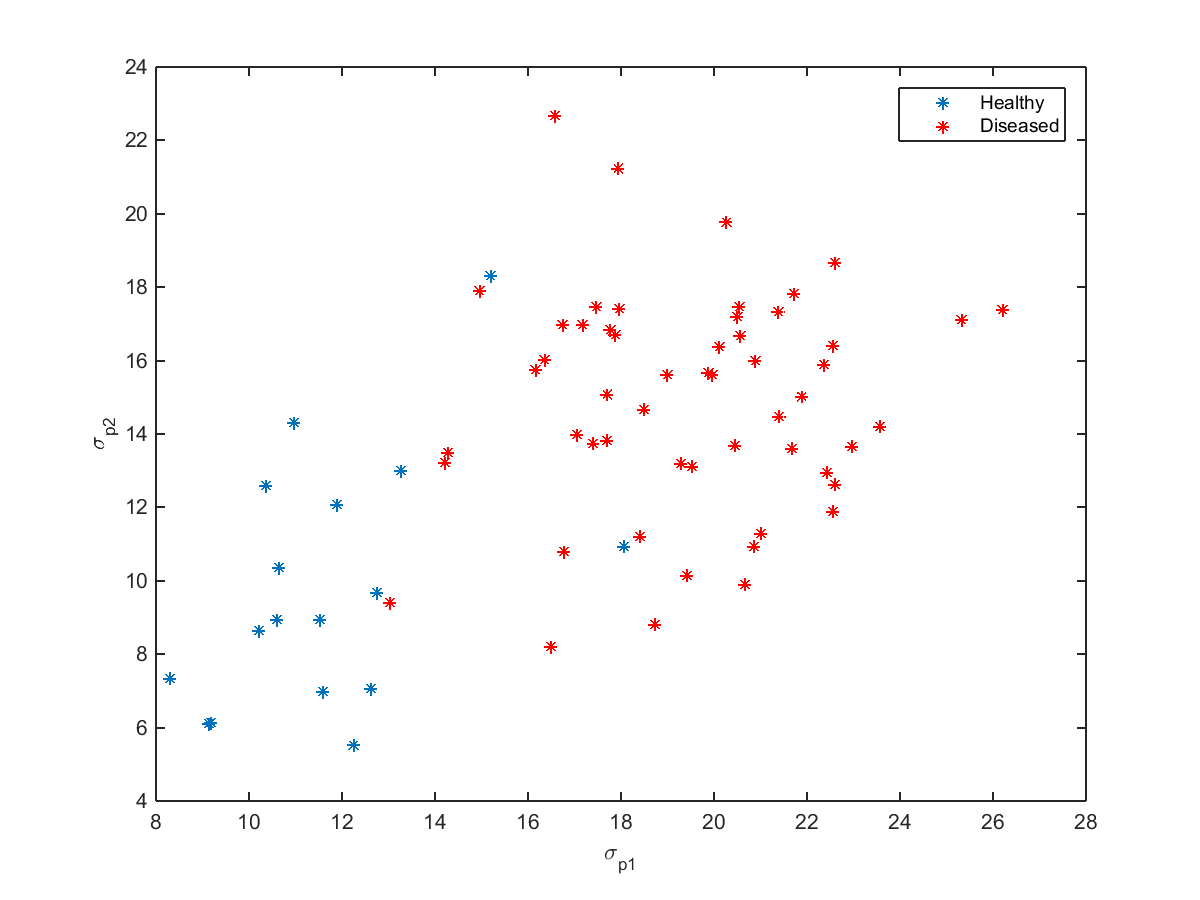

Supplement: S1 Source Code — (ZIP) [file pone.0123262.s002.zip › Results/Figure_7.png]
